# Combined and Relative Effect Levels of Perceived Risk, Knowledge, Optimism, Pessimism, and Social Trust on Anxiety among Inhabitants Concerning Living on Heavy Metal Contaminated Soil

**DOI:** 10.3390/ijerph13111076

**Published:** 2016-11-02

**Authors:** Zhongjun Tang, Zengli Guo, Li Zhou, Shengguo Xue, Qinfeng Zhu, Huike Zhu

**Affiliations:** 1 College of Economics and Administration, Beijing University of Technology, No. 100 Pingleyuan, Beijing 100124, China; 18810926839@emails.bjut.edu.cn (Z.G.); zhuhuike@emails.bjut.edu.cn (H.Z.); 2 Faculty of Business, University of Greenwich, London SE10 9LS, UK; Zl14@gre.ac.uk; 3 School of Metallurgy and Environment, Central South University, Changsha 410083, China; sgxue@csu.edu.cn; 4 School of Business, Central South University, Changsha 410083, China; zhqf150@sina.com

**Keywords:** anxiety, contaminated soil, knowledge, optimism, perceived risk, social trust

## Abstract

This research aims at combined and relative effect levels on anxiety of: (1) perceived risk, knowledge, optimism, pessimism, and social trust; and (2) four sub-variables of social trust among inhabitants concerning living on heavy metal contaminated soil. On the basis of survey data from 499 Chinese respondents, results suggest that perceived risk, pessimism, optimism, and social trust have individual, significant, and direct effects on anxiety, while knowledge does not. Knowledge has significant, combined, and interactive effects on anxiety together with social trust and pessimism, respectively, but does not with perceived risk and optimism. Social trust, perceived risk, pessimism, knowledge, and optimism have significantly combined effects on anxiety; the five variables as a whole have stronger predictive values than each one individually. Anxiety is influenced firstly by social trust and secondly by perceived risk, pessimism, knowledge, and optimism. Each of four sub-variables of social trust has an individual, significant, and negative effect on anxiety. When introducing four sub-variables into one model, trust in social organizations and in the government have significantly combined effects on anxiety, while trust in experts and in friends and relatives do not; anxiety is influenced firstly by trust in social organization, and secondly by trust in the government.

## 1. Introduction

Evidence is accumulating that exposure to chronic environmental pollution, including living on heavy metal contaminated soil (HMCS), may induce enduring anxiety [1,2,3]. Anxiety is an unpleasant emotional state characterized by tension, worry, fear, and unease [4]. Untreated and prolonged anxiety often translates into physiological stress with a wide range of effects on health and well-being [5,6]. Anxiety has detrimental effects on many aspects of immune system functioning, including viral reactivation through downregulating T-cell responses to viruses (e.g., Epstein–Barr, herpes simplex, cytomegalovirus). It may also increase inflammatory system sensitivity, including increased production of interleukin. In addition, anxiety may have aggravate physical symptoms (pain and fatigue), as well as interfere with one’s quality of life [5,6].

Because of high prevalence and negative impacts of anxiety, a growing body of studies have explored variables influencing anxiety among populations at risks [6]. One principle arising from Environmental Stress theory by Lazarus and Folkman [7] is that health consequences of environmental hazards are not only dependent on how individuals interpret and judge the hazards, but also on resources individuals have at their disposal to address the situation. According to the principle, a variable describing one kind of health consequences of environmental hazards may be an endogenous variable. Anxiety, an unpleasant emotional state, is a kind of health consequences of environmental hazards, so we used anxiety as a endogenous variable. According to the principle, exogenous variables may be divided into two groups. The first one is related to “how individuals interpret and judge the hazards”. Perceived risk refers to subjective judgments about threats posed by hazards. Therefore, perceived risk is a suitable variable for the first group. The second group is related to “resources individuals have at their disposal to address the situation”. This group can be further subdivided into two sub-groups based on resources' internal or external characteristics. The first sub-group describes internal (personal) resources. Personal resources may be divided into innate and acquired resources. Optimism/pessimism, two dispositional resources, are important innate resources. Knowledge, actual knowledge stored in one’s memory, is an important acquired resource. Therefore, we use optimism, pessimism and knowledge as variables of the first sub-group. The second sub-group describes external (social) resources. Regarding anxiety, an important social resource is information regarding how to solve HMCS problems. In today’s information society, relative to the amount of information, credibility of the information is more important. Social trust refers to a degree of an individual’s willingness to rely on information regarding how to solve HMCS problems. Therefore, social trust is a suitable variable for the second sub-group.

Individual effect levels on anxiety of perceived risk, social trust, knowledge, optimism, and pessimism have received increasing attentions over the last 10 years. Unfortunately, very few have explored combined and relative effect levels of these variables. Despite extensive knowledge of individual effect levels of these variables, very little is still known about their combined and relative effect levels on anxiety. Reliable assessment of such combined and relative effect levels seems indispensable, since people are usually influenced by mixtures of nature of risk, personal and social resources. Similarly, extant studies about an effect level of social trust on anxiety have mainly explored impact levels of individual sub-variable of social trust. Very few have investigated combined and relative effect levels of three or more sub-variables, and thus it is imperative to identify such combined and relative effect levels.

Difference in nature of risk may result in different determinants of anxiety [8,9]. Because of different nature between soil contamination and other environmental pollution, combined and relative effect levels of perceived risk, knowledge, optimism, pessimism, and social trust on anxiety may be different among inhabitants concerning living on HMCS and those living on soil with other environmental pollution, such as water and air pollution. With rapid population growth and large-scale industrialization, HMCS is becoming more and more common all over the world, especially in mainland China. Thus, it is imperative to identify those combined and relative effect levels among inhabitants concerning living on HMCS in mainland China. Similarly, it is indispensable to assess combined and relative effect levels of three or more sub-variables of social trust among inhabitants concerning living on HMCS in mainland China.

This research aims at combined and relative effect levels on anxiety of five variables comprising perceived risk, knowledge, optimism, pessimism, and social trust among inhabitants concerning living on HMCS in mainland China. A second objective is to identify combined and relative effect levels on anxiety of four sub-variables of social trust, comprising trust in the government, in social organization, in experts, and in friends and relatives in case of inhabitants concerning living on HMCS in mainland China.

## 2. Literature Review

A growing body of studies has explored variables influencing anxiety among people at risks. Because of space limitation for this article, we focused on articles on determinants of anxiety published after 2002. Table 1 illustrates the articles from authors, risks, positive and negative determinants including direct and indirect, and factors without significant effect. Michael Edelstein's seminal studies [10,11] summarized important contributions of earlier researches.

Columns 3–5 of Table 1 show that in addition to impacts of demographic variables on anxiety, influences of perceived risk, knowledge, optimism, pessimism, and social trust have received increasing attentions over the last 10 years. Column 1 of Table 1 shows that many studies aim at a wide variety of hazards, including living near incinerators or industrialized areas [1,2], facing virus infection [15,21], encountering daily hassles or fireworks disasters [14,24], and contracting different types of illnesses [16,20,23]. Despite the high number of studies on anxiety, Vandermoere [3] is the only one focusing on determinants of anxiety among inhabitants concerning living on HMCS. Factors studied by Vandermoere [3] comprise perceived participation, perceived need for decontamination, and perceived risk. To date no study has assessed combined or relative effect levels of perceived risk, knowledge, optimism, pessimism, and social trust on anxiety among inhabitants concerning living on HMCS.

Columns 3 to 5 of Table 1 show inconsistent findings about effect levels of perceived risk and knowledge on anxiety. Most researches indicate that perceived risk has a positive effect on anxiety [1,18,23], whereas Vandermoere [3] illustrates that perceived risk has no significant effect on anxiety. In terms of consequences of knowledge, Selinger et al. [20] demonstrate that it has a positive effect on anxiety, whereas an insignificant effect is illustrated by Absetz et al. [12] and Chan et al. [13] Lim et al. [9] reveal that for patients scheduled for abdominal surgery, knowledge of postoperative care can add to anxiety rather than decrease it, but acquisition of knowledge can result in reduction of anxiety for breast patient. These inconsistencies mean a need to further investigate effect levels of perceived risk and knowledge on anxiety.

## 3. The Conceptual Model Building and Research Hypothesis

**Figure 1:**
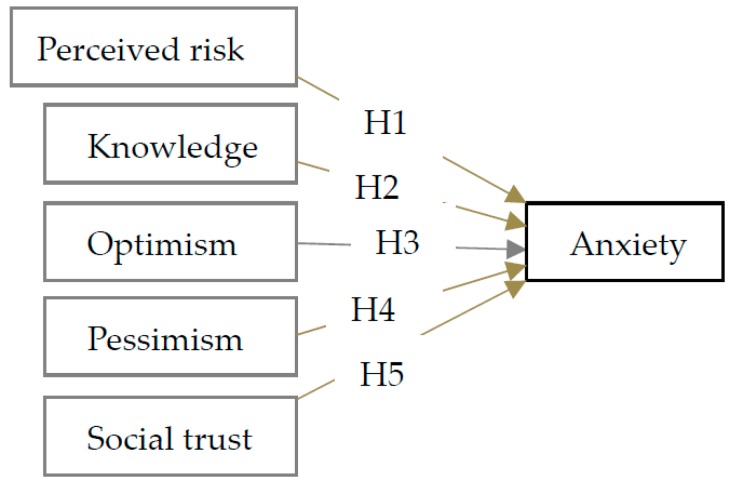
Schematic illustration of the research model 1.

Figure 1 shows the hypothesized conceptual model 1. Perceived risk, knowledge, optimism, pessimism, and social trust are exogenous variables, and anxiety as an endogenous variable.

Many scholars have defined risk perception as subjective judgments about threats posed by hazards [27]. Similarly, we define perceived risk from HMCS as subjective judgments about both attributes of heavy metal soil contamination and potentially environmental and biological harms posed by the contamination. Weber et al. [28] argue that people living in contaminated areas perceive the risk from HMCS as being high. Grasmuck and Scholz [29] reveal that people living in close proximity to a metal-processing plant perceive the risk to themselves as medium, and perceive the risk for others as high. HMCS may cause health risks in a long-term exposure scenario [28,29]. Long-term consumption of groundwater and food grown on HMCS are the most critical paths of pollution [28,29]. Several studies have confirmed that perceived risk significantly increases anxiety due to hazards other than living on HMCS, such as living close to an incinerator [1] and working in the Norwegian offshore oil and gas industry [18]. These findings offer indirect evidences to support hypothesis concerning increased anxiety from perceived risk among inhabitants due to living on HMCS. Hypothesis one (H1) is that perceived risk has a positive effect on anxiety.

Any hazard may result in negative outcomes or losses. For inhabitants concerning living on HMCS, losses may include harms to their health or to the environment. In this research, we define knowledge as actual knowledge stored in one’s memory on whether HMCS may result in environmental or biological harms. According to Bibb [30], negative messages, such as knowledge of some forms of losses, may promote negative thinking, and in turn trigger anxiety. Thus, knowledge of harms resulting from HMCS may have a positive effect on anxiety among inhabitants concerning living on HMCS. Some researchers have empirically demonstrated positive associations between knowledge and anxiety due to illness, such as inflammatory bowel disease [20] and abdominal surgeries [9]. Hypothesis two (H2) is that knowledge has a positive effect on anxiety.

In this research, we approach optimism as a dispositional resource, “as a generalized tendency to expect positive outcomes, as the belief that good rather than bad things will happen in a person’s life”, and as “the degree to which people expect the best in uncertain times” [31]. According to Carver and Scheier [32], optimists should display confidence and persistence when encountering stressful situations. Thus, optimism may help individuals to better adjust to negative situations, such as living on HMCS. Compared with pessimists, optimists may use adaptive and positive thinking to deal with risks, including accepting reality and seeing it in a positive light [31]. Even though optimists may accept that they cannot control heavy metal soil contamination, they might perceive that information gives them a better opportunity to deal with consequences resulting from the contamination. In contrast, pessimism may lead people to make global, internal, and stable causal attributions to negative events, which in turn produce negative mental health effects [33] and even long-term health effects [34]. As a result, optimism/pessimism may have an inverse/proportional effect on anxiety among inhabitants concerning living on HMCS. Optimism has been illustrated to be significantly associated with negative anxiety among Israeli residents who experienced missile attacks during the Gulf War [35]; Chinese cervical cancer patients [6]; and women seeking genetic counseling for breast cancer [19,25]. German urogenital cancer patients with low levels of optimism and high levels of pessimism are found at risk of higher levels of anxiety [26], as are Norwegian female patients one year following breast cancer surgery [22]. In a longitudinal study of the Enschede Fireworks Disaster, Velden et al. [24] have demonstrated that pessimistic victims are more at risk of severe anxiety symptoms than optimists are. Hypothesis three (H3) is that optimism has a negative effect on anxiety and Hypothesis four (H4) is that pessimism has a positive effect on anxiety.

Social trust refers to a degree of an individual’s willingness to rely on information regarding how to solve HMCS problems, where this information may be obtained from the government, social organizations, experts, or friends and relatives. Control/alienation theory by Ross and Mirowsky [36] states that anxiety results from an inability to address a problem. To compensate for this inability, an affected person may seek others’ support in order to gain control over the issue. Social support might serve as a protective factor in development of anxiety [37,38]. Some authors [39] have stressed the importance of social trust. When people sense a lack of personal control over hazards, they prefer to attend to only messages that come from sources they perceive as trustworthy. Trust has a significantly negative relationship with anxiety concerning hazards such as SARS crisis [15], food safety [8], and cancer [16]. These findings offer indirect evidences to support hypothesis concerning decreasing anxiety via social trust among inhabitants due to living on HMCS. Hypothesis five (H5) is that social trust has a negative effect on anxiety.

**Figure 2:**
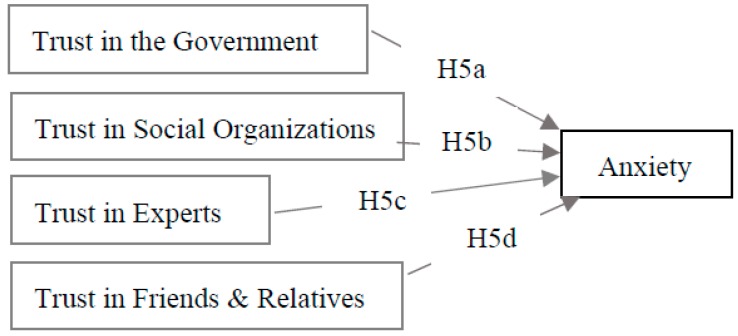
Schematic illustration of the research model 2.

Regarding HMCS, inhabitants are incapable of dealing with the contamination and may sense a lack of personal control over it; thus, they may prefer messages from authorities and experts. In addition, information from social organizations, friends, and relatives is usually used by Chinese people [40]. Thus, in this research, social trust consists of four sub-variables: trust in the government, in social organizations, in experts, and in friends and relatives. Hypothesis five (H5) then may be divided into four sub-hypotheses (Figure 2):

Hypothesis H5a is that trust in the government has a negative effect on anxiety.

Hypothesis H5b is that trust in social organizations has a negative effect on anxiety.

Hypothesis H5c is that trust in experts has a negative effect on anxiety.

Hypothesis H5d is that trust in friends and relatives has a negative effect on anxiety.

## 4. Method

### 4.1. The Surveyed Areas

Contaminated soil is a common environmental problem all over the world. A major kind of such contamination is caused by heavy metals. This kind of contamination has its source mainly in emissions from heavy metal mining and/or processing plants. A perfect example is some areas in Xiangjiang River basin of Hunan Province, China. The River rises in Haiyang Mountains of Kwangsi Chuang Autonomous Region, and flows through several industrial and mining areas (e.g., Shuiguoshan, Hengyang, and Zhuzhou) of Hunan province on its way to Dongting Lake. The river has a length of about 856 km (with 670 km in Hunan Province) and the area of its drainage basin is 94,660 km^2^. The basin contains a number of large deposits of nonferrous metals, which have stimulated an extensive metallurgical industry in the region. Soils in several areas surrounding these metal mining/processing plants have been contaminated by copper, cadmium, lead, and/or zinc [41].

We chose villages from Xiangjiang River basin in Hunan Province based on two criteria: in close proximity to metal-processing/metal-mining area and at least one incident caused by HMCS in the area within the last 10 years. We thus selected four villages: (1) Guangfa, Jiahe, Chenzhou; (2) Hanglang, Jiahe, Chenzhou; (3) Majiahe; Zhuzhou, and (4) Songbai, Changning. These four villages share similar demographic, economic, cultural, and ethnic characteristics. Moreover, we found no significant differences among the villages regarding residents’ age, gender, and education level.

### 4.2. Sample and Procedure

We applied three-stage sampling to obtain reliable and valid data [42]. In stage 1, we selected 15 inhabitants living on the surveyed areas, and who are familiar with HMCS, via purposeful sampling, and conducted interviews using open-ended questions during March 2012. This stage is necessary to construct a set of items that are appropriate to our target sample. Through the interviews we collected information about attributes and potential outcomes of HMCS. The information provide a basis for our questionnaire design.

After questionnaire designed, we conducted a pilot study, the second stage, in April 2012. This stage aims at verifying that individuals with limited education can answer the questionnaire, including verifying its wording, response formats, and clarity of instructions. We purposefully tested a sample containing 15 inhabitants from the surveyed areas, five of whom had received only primary education. We altered the questionnaire according to their feedback.

In stage 3, we conducted on-site surveys via door-to-door sampling in June and August 2012. For each village investigated, we started a sampling process from a family nearest to the plant that had caused a soil-contamination incident, and gradually spread out until we had either distributed about 150 questionnaires, or reached a distance of approximately one kilometer from the plant. We selected one family among every three and asked one adult from each to complete the questionnaire. A total of 600 questionnaires were distributed and 499 valid questionnaires completed, resulting in a response rate of 83.2%. Table 2 summarizes the respondent demographics.

### 4.3. Questionnaire Design

We constructed questionnaire items (see Table A1 in Appendix A) using validated items from prior studies and information from interviews at the first stage of this research. Specifically, we adapted the State Anxiety Scale [43] to measure level of anxiety resulting from participant experiences, as this scale is theoretically based and structurally valid, suitable for field use, and easy to circulate and evaluate [4]. The scale comprises 20 statements describing emotional states. The score for each individual item varies from 1 to 5, so that the total score ranges from 20 to 100. A higher score denotes a higher level of anxiety (20–40 points = minimal anxiety; 41–60 points = moderate anxiety; 61–80 points = intense anxiety; 81–100 points = maximal anxiety). Factor loadings of the 20 items range from 0.419 to 0.714, and Cronbach’s alpha is 0.849 (Appendix A).

Similarly, we adapted the Revised Life Orientation Test (LOT-R) to measure optimism/pessimism because it shows good internal consistency, test–retest reliability, and convergent and discriminant validity [33]. The LOT-R includes 10 items to measure optimism and pessimism; six items (OP1–OP6) measure optimism, and four (PE1–PE4) measure pessimism. Factor loadings of optimism items range from 0.484 to 0.711, and Cronbach’s alpha is 0.738; factor loadings of pessimism items range from 0.481 to 0.733, and Cronbach’s alpha is 0.757 (Appendix A).

We designed 12 items to measure perceived risk due to HMCS by referring to measurements by Slovic [44], and information from interviews at the first stage of this research. The first seven (PR1–PR7) are designed to measure attributes of contamination and the following five (PR8–PR12) to measure potential harms according to participants’ subjective feelings. We did not design any item to measure perceived likelihood of events caused by HMCS, as we selected respondents from areas where such contamination had occurred. All items are similar to those of Slovic [44] and adapt to the situation of risk arising from HMCS. Factor loadings of the 12 items range from 0.485 to 0.680 and Cronbach’s alpha is 0.851 (Appendix A).

As we define social trust as a degree of one’s willingness to rely on information obtained from (1) the government; (2) social organizations; (3) experts; and (4) friends and relatives, we designed four items to measure social trust. Factor loadings of the four items range from 0.610 to 0.765 and Cronbach’s alpha is 0.814 (Appendix A).

Knowledge on soil contamination may include that on transmission paths from contaminated soil to the human body, environmental and biological harms, etc., and thus it may be necessary to assess knowledge not from an overall point of view but from several different aspects. However, to our knowledge, in the field of soil contamination only Grasmuck and Scholz [29] have developed an instrument to measure knowledge in this way. They developed three items to measure actual knowledge from three aspects: substances that cause health risks, the most relevant route for human intake of heavy metals, and vegetables or fruits whose regular consumption should be avoided. These items do not assess knowledge on environmental and biological harms, while we define knowledge as actual knowledge on environmental and biological harms. Hence, we developed items to measure knowledge based on information from interviews at the first stage of this research. During the interviews we found that inhabitants believed there are five main kinds of harms: (1) water pollution; (2) reduced soil quality; (3) endangered human health; (4) endangered livestock; and (5) endangered crops. We then designed five items to measure actual knowledge from an objective perspective, rather than from participants’ subjective feelings. Factor loadings of the five items range from 0.486 to 0.722 and Cronbach’s alpha is 0.777 (Appendix A).

For the aforementioned measures except knowledge, a five-point Likert scale was used, with anchors ranging from 1 = strongly disagree to 5 = strongly agree, with 3 representing neutral. For measures of knowledge, a three-point Likert scale was used, with anchors ranging from 1 = no to 3 = yes, with 2 representing unknown. Our surveys were conducted in Chinese. Items to measure anxiety, optimism, and pessimism were translated to meet local requirements. A panel of three Chinese academic experts on environment management engaged in an iterative process to execute the translation. All translation disagreements were resolved via a group process involving all three experts. A professional translator then back translated the Chinese version into English to verify equivalence of the two versions. In the actual surveys completed by respondents, all items were randomly sequenced. The last part of the questionnaire collected information on each of respondent’s age, gender, education level, and occupation. As mentioned earlier, as for a final check, the Chinese questionnaire was reviewed and tested in the second stage.

### 4.4. Statistical Analysis

All analyses were conducted using SPSS version 19.0 with a two-tailed probability value of 0.05 considered statistically significant. Descriptive statistics of demographics and all variables are indicated with means and standard deviations, as appropriate. To assess discriminant validity of all scales, KMO values were extracted, and followed by Bartlett’s test of sphericity and principal components analyses. We analyzed reliability of all scales using Cronbach’s alpha values. Variations in anxiety were examined with respect to demographics using independent sample *t*-test and one-way ANOVA. Pearson’s correlation coefficients resulting from bivariate correlation analyses were used to examine correlations among all variables. Linear regression analysis was used to explore individual effect levels of each exploratory variable and each sub-variable of social trust on anxiety. We used multivariate stepwise regression to investigate combined and relative effect levels on anxiety of every explanatory variable by examining changes in the *R*^2^*_adjusted_* and beta coefficients as explanatory variables were added to each specification. Only variables remaining significantly associated with anxiety with *p*-values of < 0.05 were kept in the final model. Moreover, tolerance and variance inflation factor (VIF) values were used to check for multicollinearity. Similarly, we used multivariate stepwise regression to assess combined and relative effect levels of four sub-variables of social trust. Multiple linear regression analysis by enter method was used to explore combined and interactive effect levels on anxiety rising from knowledge, together with other explanatory variables, respectively. Similarly, we used multiple linear regression analysis by enter method to explore combined effect levels of four sub-variables of social trust.

## 5. Results

The mean (±SD) scores of anxiety are 72.05 ± 8.64. The prevalence of moderate, intense, and maximal anxiety is 12.1%, 78.6%, and 18.9%, respectively. An independent samples *t*-test fails to indicate statistically significant relationships between anxiety and gender (F = 2.586, *p* = 0.108). Results from one-way ANOVA reveal that anxiety is significantly related to age (F = 7.599, *p* < 0.001) and to occupation (F = 5.736, *p* < 0.001), but not to education level (F = 1.635, *p* = 0.164).

Pearson’s correlation coefficients by bivariate correlation analysis, as shown in Table 3, reveal that anxiety is positively related to perceived risk and pessimism, with statistical significance (*p* < 0.01), and positively related to knowledge, but without statistical significance (*p* > 0.05). Significantly negative associations (*p* < 0.01) between social trust, optimism, and anxiety are observed.

When considered individually using linear regression analyses, social trust, perceived risk, pessimism, and optimism are found to have significant effects on anxiety, while a significant effect of knowledge on anxiety is not noted (Table 4). Stepwise multiple regression analysis confirms that when introduced together into one model (Model 12, Table 5), social trust, perceived risk, pessimism, knowledge, and optimism have significantly combined effects on anxiety, while age and occupation are excluded from the regression model because they provide a comparatively small contribution to explain the variance. The effects of social trust and optimism are negative and those of perceived risk, pessimism, and knowledge are positive when analyzed individually, and when introduced into one model.

Although a positive influence of knowledge on anxiety is significant when introduced into two combined models 11 and 12 (Table 5), this effect becomes non-significant when analyzed individually (Model 6, Table 4). Furthermore, the effect of knowledge on anxiety is significant when introducing knowledge into one model together with social trust and pessimism, respectively (Table 6). If analyzed when introducing knowledge together with perceived risk or optimism into one model, this effect is non-significant. Comparisons of Table 4 and Table 6 show that effect coefficients of social trust and pessimism on anxiety have increased after introducing knowledge. The variance in anxiety is more clearly explained after introducing knowledge into a combined model with social trust and pessimism, respectively. Furthermore, social trust and knowledge have a significantly and positively interactive effect on anxiety; pessimism and knowledge have a significantly and negatively interactive effect on anxiety (Table 7).

Due to significant correlations between any two independent variables (Table 3) and an insignificant correlation between knowledge and anxiety (Table 4), an important portion of predictive value of either of independent variables, except knowledge (Models 3–7, Table 4) was displaced by introducing the respective other into Model 12 (Table 5). These effects cannot be attributed to multicollinearity of the variables, since with detection-tolerance values of 0.780–0.997 (Table 5) and VIF of 1.282–1.003, indicators for multicollinearity are respectively well above and below recommended thresholds for tolerance >0.20–0.10 and VIF < 5–10.

Comparisons of different stepwise regression models (Models 8–12, Table 5) show that, by itself, social trust explains 27.1% of the variance in levels of anxiety in the initial step (Model 8). When adding perceived risk to the analysis, the explained variance is increased significantly to 38.8% in the second step (Model 9). However, the 11.7% rate of increase is far less than the 27.1% noted in the initial step. Similarly, from the second step (Model 9) to the third (Model 10), to the fourth (Model 11), and to the fifth (Model 12), each rate of increase in the explained variance is far less than the amount explained by social trust, and descended step by step. Additionally, 45.3% of the variance is explained by five independent variables in the fifth step (Model 12). Four variables are added from the initial to the fifth step, but only 18.2% more of the variance can be explained, which is obviously less than 27.1%. Thus, of all the explanatory variables, social trust plays a central role in predicting anxiety. Anxiety is influenced firstly by social trust, and secondly by perceived risk, pessimism, knowledge, and optimism.

When considered individually via linear regression analyses, each of four sub-variables of social trust has a significantly negative effect on anxiety (Models 19–22, Table 8). However, stepwise multiple regression analyses confirm that when introduced together into one model (Model 24, Table 8), trust in social organizations and in the government have significantly negative effects on anxiety, while trust in experts and in friends and relatives are excluded from the regression model because they have contributed relatively little to the explanation of variance. Results of linear regression analyses of all sub-variables by enter method also confirm that trust in social organizations and in the government have significantly negative effects on anxiety, while trust in experts and in friends and relatives have insignificantly negative effects (Model 25, Table 8).

Stepwise multiple regression analyses of four sub-variables of social trust (Model 24, Table 8) show that effects of trust in social organizations and in the government cannot be attributed to the variables’ multicollinearity, since the detection-tolerance values of 0.541–0.945 (Table 8) and VIF of 1.848–1.058 are respectively well above and below recommended thresholds. Comparisons of different stepwise regression models (Models 23 and 24) show that, by itself, trust in social organizations can explain 24.3% of the variance in levels of anxiety in the initial step (Model 23). When adding trust in the government to the analysis, the explained variance is increased significantly to 28.6% in the second (final) step (Model 24). Additionally, in Model 24 the effect level of trust in social organizations is significantly higher than that of trust in the government. Thus, of the four sub-variables, trust in social organizations plays a central role in predicting anxiety.

## 6. Discussion

### 6.1. Theoretical Implications

This research confirms intense anxiety among inhabitants concerning living on HMCS, which is in line with Vandermoere [3], who also argues that inhabitants living on polluted soil have high scores for anxiety. However, our results deviate from Vandermoere [3] in the absence of a relationship between perceived risk and anxiety. In this research, perceived risk has a significantly positive effect on anxiety. An explanation for this difference may be that respondents in Vandermoere’s [3] study have felt able to control pollution routes and then can distinguish between risks and hazards, while respondents in this research are unable to control routes. Our conclusion is in line with that of Lima [1], Nielsen et al. [18], and Vansenne et al. [23], who have concluded that perceived risk has a significantly positive effect on anxiety.

An important result of this research is that social trust, perceived risk, pessimism, knowledge, and optimism account for a moderate proportion of variance in anxiety (45.3%, Model 12). More importantly, the five variables as a whole have stronger predictive values than each individually, indicating that the combined measure of the five variables may be more effective than using any individual variable to predict anxiety. This provide an evidence of application of Environmental Stress theory to prediction and understanding of anxiety of inhabitants concerning living on HMCS. Furthermore, social trust, perceived risk, and pessimism can explain 42.0% of variance in anxiety (Model 10), showing that a combined measure of the three variables may be more cost-efficient than using all five variables or one variable individually to predict anxiety.

Our results show that of all independent and socio-demographic variables, social trust has the strongest effect level on anxiety. An explanation for this may be that the nature of social trust differs from that of other variables from a resource perspective. Resources are strengths or traits that an individual has at her/his disposal to cope with stress [45], and can be divided into two categories: personal and social. Social trust is seen as a critical social resource [46]. Personal resources may be divided into affective, cognitive, and instrumental. Affective resources include all emotions that individuals have within themselves that enable them to cope with unusual or new events, such as uncertainty. Optimism and pessimism are seen as positive and negative affective resources, respectively. Instrumental resources result from an individual’s demographic variables. Cognitive resources relate to individuals’ ideologies, political orientation, and cognitive abilities, which may prove useful in understanding usual phenomena [45]. Education enables individuals to understand complicated situations and solve problems [45]. Education level and knowledge are seen as cognitive resources. Compared with social resources, individual resources to avert environmental threats are generally low. Of all the independent and socio-demographic variables, therefore, social trust may play the strongest role in anxiety.

Regarding an individual influence of knowledge on anxiety, we argue for the same insignificant conclusion as Absetz et al. [12], that risk factor knowledge has no significant effect on anxiety. Similarly, Chan et al. [13] provide empirical evidence that anxiety in female patients is unrelated to their levels of knowledge. A possible reason for the insignificant relations proposed by Chan et al. [13] is that anxiety is jointly influenced by knowledge and other factors. The studies by Absetz et al. [12] and Chan et al. [13] did not examine others’ effects on anxiety as they focused on effects of knowledge (and experience), while we examined effects of social trust, perceived risk, pessimism, and optimism alongside knowledge. One interesting finding is that although knowledge does not have individually and significantly direct effects on anxiety, it has combined significant effects on anxiety together with social trust and pessimism, respectively. Similar findings regarding insignificant individual effects but with significant combined effects have been found by Sapp and Bird [8]; in their empirical study on determinants of consumers’ food safety anxiety, they argue that environmentalism has no significant direct effect on anxiety, but has significant combined effects together with social-demographic variables. Unfortunately, Sapp and Bird [8] do not give reasons for their findings. A possible reason may be interactive effects on anxiety between different variables. In particularly, a possible reason for our findings may be that knowledge has interactive effects on anxiety with social trust and pessimism. Furthermore, the interactive effects have been confirmed in this research.

Another interesting finding of this research is that of four sub-variables of social trust, each has an individual, significant, and negative direct effect on anxiety; however, when introducing all four sub-variables together into one model, trust in social organizations and in the government have significant negative effects on anxiety, while trust in experts and in friends and relatives have no significant effects. An explanation may be that because social organizations and the government are collective organizations rather than individuals, respondents may perceive these groups’ abilities to deal with HMCS problems to be higher, compared to experts or friends and relatives. Similarly, respondents may perceive messages provided by social organizations and government to be more trustworthy than those by experts or friends and relatives. Higher perceived abilities and more trustworthy messages may result in less anxiety, according to control/alienation theory [36].

### 6.2. Practical Implications

This research confirms intense anxiety among inhabitants concerning living on HMCS, suggesting that it is necessary for the local government and social organizations to help the inhabitants to reduce their anxiety.

Social trust, perceived risk, pessimism, knowledge, and optimism have significantly combined effects on anxiety and account for 45.3% of variance in anxiety. The five variables as a whole have stronger predictive values than each individually. Thus, the local government and social organizations should adopt comprehensive measures to reduce inhabitants’ anxiety, rather than a single measure. Furthermore, this research shows that social trust, perceived risk, and pessimism explain 42.0% of variance in anxiety. A combined measure of the three variables may be more cost-efficient than using all five variables or one variable individually to predict anxiety. These suggest that the comprehensive measures relating to social trust, perceived risk, and pessimism should be emphasized.

Anxiety is influenced firstly by social trust. Furthermore, when introducing four sub-variables into one model, trust in social organizations and in the government have significant negative effects on anxiety, while trust in experts and in friends and relatives have no significant effects. Thus, in order to reduce anxiety among inhabitants concerning living on HMCS, the most important job by the local government and social organizations is to increase credibility of the information provided by them, regarding how to solve HMCS problems. This research indicates that anxiety is influenced secondly by perceived risk with a significantly positive effect. These suggest that in the absence of an effective method to remove heavy metals from contaminated soils at present, inhabitants should be educated about methods to control pollution routes and to distinguish between risks and hazards. Our results also highlight that based on effect levels on anxiety of social trust, perceived risk, pessimism, knowledge, and optimism, pessimism ranks the third within the variables, whereas optimism takes the sixth position. Therefore, compared with optimists, pessimists should be given more concern.

### 6.3. Limitation and Future Research

In interpreting findings of this research, we should consider its limitations. First, the sample is regional. Second, no replication using an additional sample is included. Third, the variable choices could be criticized, in that other variables should be added because 54.7% of the variance in anxiety (Model 12, Table 5) could not be explained. For example, a potential limitation is respondents’ lack of a sense of participation in consultations over HMCS problems, lack of environmental annoyance, and lack of trust in polluting enterprises. Vandermoere [3] argues that mental health burdens from living on contaminated soil can be induced by a lack of feeling of participation; Lima [1] highlights an habituation effect on mental health for those living closer to an incinerator; and Lopez-Navarro et al. [47] confirm regarding industrial risks, it is essential to distinguish between trust in companies that make up an industry. Because of the above limitations, there is a need to conduct research in multiple areas to confirm the results, and add other variables and test their corresponding effects to explain a greater proportion of the variance in anxiety.

## 7. Conclusions

This research effort represents an attempt to address a noticeable gap in literature on combined and relative effect levels on anxiety of: (1) perceived risk, knowledge, optimism, pessimism, and social trust; and (2) four sub-variables of social trust among inhabitants concerning living on HMCS on the basis of survey data from 499 Chinese respondents.

Regarding combined effect levels on anxiety of perceived risk, knowledge, optimism, pessimism, and social trust, although knowledge does not have individual, significant, and direct effects on anxiety, knowledge has significant, combined, and interactive effects on anxiety together with social trust and pessimism, respectively, while does not with perceived risk and optimism. Furthermore, social trust, perceived risk, pessimism, knowledge, and optimism have significantly combined effects on anxiety. The five variables as a whole have stronger predictive values than each one individually, which provides evidence of application of Environmental Stress theory to prediction and understanding of anxiety of inhabitants concerning living on HMCS. A combined measure of social trust, perceived risk, and pessimism may be more cost-efficient than using all five variables or one variable individually to predict anxiety. In terms of relative effect levels of these five variables, anxiety is influenced firstly by social trust and secondly by perceived risk, pessimism, knowledge, and optimism. Social trust plays the strongest role in anxiety within these five variables.

Regarding combined effect levels on anxiety of four sub-variables of social trust, although each of four sub-variables of social trust has an individual, significant, and negative effect on anxiety, trust in social organizations and in the government have significantly combined effects on anxiety, while trust in experts and in friends and relatives do not. In terms of relative effect levels, anxiety is influenced firstly by trust in social organization, and secondly by trust in the government.

## Figures and Tables

**Table 1:** Review of determinants of anxiety among people at risks.

Authors Factors	Risks	Positive Factors (Direct & Indirect)	Negative (Direct & Indirect)	Factors with No Significant Effect
Absetz et al. [12]	BC	-	-	EX and KN
Chan et al. [13]	CA	-	-	KN
Chen and Hong [14]	DS	IID	-	IU
Cheung and Tse [15]	SARS	-	TIG, TIM	-
Hinnen et al. [16]	CA	-	TIP	-
Hou et al. [17]	CC	-	FI, FC, FE	-
Lev-Wiesel and Kaufman [4]	UI	DU	PT, SSF	SSD, MQ
Lim et al. [9]	BAS	-	-	KN
	BS	-	KN	-
	AS	KN	-	-
Lima [1]	IN	EL, PR, IPE	-	AG, EA
Marques and Lima [2]	IA	AP	PIA	IIR
Nielsen et al. [18]	OOG	PR, WB	SE	-
Norman and Brain [19]	FHBC	-	DO	-
Sap and Bird [8]	FS	ST, ES		EN, FM, PC
Selinger et al. [20]	CD and UC	KN	-	-
Taha et al. [21]	H1N1	IU, AVS	AVC	-
Schou et al. [22]	BCS	EL, DP, FA	DO	-
Vandermoere [3]	SC	-	PP, PND	GE, AG, PR
Vansenne et al. [23]	RM or PSQ	PR	-	-
Velden et al. [24]	FD	DP	DO	-
Wiering et al. [25]	CA	-	DO	-
Yang et al. [6]	CA	-	DO, HP	GS
Zenger et al. [26]	UGC	DP	DO	-

Note: BC = breast cancer; EX = experience; KN = knowledge; CA = cancer; DS = daily hassles; IID = interaction between intolerance of uncertainty and daily hassles; IU = intolerance of uncertainty; SARS = severe acute respiratory syndrome; TIG = trust in the government; TIM = trust in medical institutions; TIP = trust in physicians; CC = colorectal cancer; FI = family intimacy; FC = family commitment; FE = friendship; UI = unemployed immigrants in the context of military conflict and political uncertainty; DU = duration of unemployment; PT = potency; SSF = social support from family; SSD = social support from friends; MQ = marital quality; BAS = breast and abdominal surgery; BS = breast surgery; AS = abdominal surgery; IN = incinerator; EL = education level; PR = perceived risk; IPE = interaction between perceived risk and environment annoyance; AG = age; EA = environment annoyance; IA = industrialized areas; AP = air pollution; PIA = perception of industrial area; IIR = interaction between industrial perception and area of residence; OOG = offshore oil and gas industry; WB = workplace bullying; SE = self-esteem; FHBC = family history of breast cancer; DO = dispositional optimistic; FS = food unsafety; ST = social trust; ES = environmentalism and social-demographic variables; EN = environmentalism; FM = familiarity with food safety issues; PC = perceived control; CD = Crohn’s disease; UC = ulcerative colitis; AVS = appraisals of viral stress; AVC = appraisals of viral control; BCS = breast cancer surgery; DP = dispositional pessimistic; FA = fatalism; SC = soil contamination; PP = perceived participation; PND = perceived need for decontamination; GE = gender; RM = recurrent miscarriage; PSQ = poor semen quality; FD = fireworks disaster; HP = hope; GS = general self-efficacy; UGC = urogenital cancer.

**Table 2:** Survey respondent profiles.

Profile	Category	Count	Proportion (%)
Gender	Male	213	42.7
	Female	286	57.3
Age	18–27 Years Old	129	25.9
	28–37 Years Old	117	23.4
	38–47 Years Old	142	28.5
	48–57 Years Old	67	13.4
	Over 57 Years Old	44	8.8
Education Level	Primary School	25	5.0
	Junior High School	177	35.5
	Senior High School	241	48.3
	College or University	54	10.8
	Master’s or Higher	2	4.0
Occupation	Peasantry	117	22.2
	Blue-Collar	66	13.2
	Student	33	6.6
	White-Collar	6	1.2
	Self-Employed	101	20.2
	Unemployed	47	9.4
	Retiree	35	7.0
	Other	100	20.0

**Table 3:** Variable correlation coefficients.

Variable	AN	PR	ST	KN	OP
PR	0.444 **	-	-	-	-
ST	−0.431 **	−0.263 **	-	-	-
KN	0.033 ^NS^	0.139 **	0.360 **	-	-
OP	−0.227 **	0.091 *	0.201 **	0.096 *	-
PE	0.372 **	0.178 **	−0.465 **	0.192 **	−0.178 *

Note: ** significant at *p* < 0.01; * significant at *p* < 0.05; ^NS^ Non-significant; AN = anxiety; KN = knowledge; OP = optimism; PE = pessimism; PR = perceived risk; ST = social trust.

**Table 4:** Summary of individual effect levels on anxiety by linear regression analyses of age, occupation, social trust, perceived risk, pessimism, knowledge, and optimism, respectively.

Model	*R*^2^*_adjusted_*	F	Standardized Beta of Variables						
			AG	OC	ST	PR	PE	KN	OP
1	0.007	4.632	−0.096 *	-	-	-	-	-	-
2	0.007	4.658	-	−0.096 *	-	-	-	-	-
3	0.270	187.708	-	-	−0.521 ***	-	-	-	-
4	0.218	141.406	-	-	-	0.468 ***	-	-	-
5	0.179	110.429	-	-	-	-	0.425 ***	-	-
6	0.001	0.455	-	-	-	-	-	0.030 ^NS^	-
7	0.044	24.194	-	-	-	-	-	-	−0.214 ***

Note: *** significantat *p* < 0.001; * significant at *p* < 0.05; ^NS^ Non-significant; AG = age; OC = occupation.

**Table 5:** Summary of combined and relative effect levels on anxiety by stepwise regression analysis of age, occupation, social trust, perceived risk, optimism, knowledge, and pessimism.

**Model**	***R*^2^*_adjusted_***	**F**	***ΔR*^2^*_adjusted_***	**Standardized Beta of Variables**				
				**ST**	**PR**	**PE**	**KN**	**OP**
8	0.271	187.891		−0.522 ***-	-	-	-	-
9	0.388	160.205	0.117	−0.428 ***	0.356 ***	-	-	-
10	0.420	122.527	0.032	−0.335 ***	0.343 ***	0.208 ***	-	-
11	0.444	101.505	0.024	−0.406 ***	0.299 ***	0.216 ***	0.176 **	-
12	0.453	84.284	0.009	−0.392 ***	0.299 ***	0.205 **	0.178 **	−0.101 **
**Model**	**Detection-Tolerance Values of Excluded Variables**							
	**AG**		**OC**	**ST**	**PR**	**PE**	**KN**	**OP**
8	0.997		0.971	-	0.931	0.783	0.870	0.960
9	0.988		0.971	-	-	0.780	0.812	0.960
10	0.985		0.971	-	-	-	0.810	0.951
11	0.985		0.965	-	-	-	-	0.950
12	0.976		0.964	-	-	-	-	-

Note: *** significant at *p* < 0.001; ** significant at *p* < 0.01.

**Table 6:** Summary of combined effect levels on anxiety by multiple linear regression analyses of knowledge and social trust, perceived risk, pessimism, optimism, respectively.

Model	*R*^2^*_adjusted_*	F	Standardized Beta of Variables				
			ST	KN	PR	PE	OP
Combined Effect Level of ST and KN							
13	0.324	121.700	−0.612 ***	0.251 ***	-	-	-
Combined Effect Level of PR and KN							
14	0.218	71.104	-	0.037 ^NS^	0.474 ***	-	-
Combined Effect Level of PE and KN							
15	0.190	59.929	-	0.115 ***	-	0.447 ***	-
Combined Effect Level of OP and KN							
16	0.045	12.803	-	0.052 ^NS^	-	-	−0.219 ***

Note: *** significant at *p* < 0.001; ^NS^ Non-significant.

**Table 7:** Summary of combined and interactive effect levels on anxiety by multiple linear regression analyses of knowledge and social trust, pessimism, respectively.

Model	*R*^2^*_adjusted_*	F	Standardized Beta of Variables				
			ST	KN	PE	KN × ST	KN × PE
Combined and Interactive Effect Levels of ST and KN							
17	0.335	85.489	−0.616***	0.200 ***	-	0.122 **	-
Combined and Interactive Effect Levels of PE and KN							
18	0.198	42.513	-	0.093 *	0.475 ***	-	−0.108 *

Note: *** significant at *p* < 0.001; ** significant at *p* < 0.005; * significant at *p* < 0.05.

**Table 8:** Summary of individual effect levels by linear regression analyses, combined and relative effect levels by stepwise regression analysis of trust in the government, in social organizations, in experts, and in friends and relatives on anxiety.

Model	*R*^2^*_adjusted_*	F	Standardized Beta of Variables			
			TIG	TIS	TIE	TIF
Individual Effect Levels by Linear Regression Analyses						
19	0.205	130.872	−0.454 ***	-	-	-
20	0.244	162.551	-	−0.494 ***	-	-
21	0.151	90.783	-	-	−0.391 ***	-
22	0.028	15.342	-	-	-	−0.172 ***
Combined and Relative Effect Levels by Stepwise Regression Analysis						
23	0.243	162.551	-	−0.494 ***	-	-
24	0.286	101.899	−0.256 ***	−0.349 ***	-	-
Detection-Tolerance Values of Excluded Variables						
23	-		0.679	-	0.560	0.945
24	-		-	-	0.541	0.942
Combined Effect by Multiple Linear Regression Analyses (Enter Method)						
25	0.287	51.737	−0.244 ***	−0.307 ***	−0.058 ^NS^	−0.044 ^NS^

Note: *** significant at *p* < 0.001; ^NS^ Non-significant; TIS = trust in social organizations; TIE = trust in experts; TIF = trust in friends and relatives.

## Appendix A

**Table A1:** Items to measure anxiety, optimism, pessimism, perceived risk, social trust, and knowledge.

Variable/Question/Alpha Value	Factor Loadings
Anxiety (AN) (alpha = 0.849)	
Living on Heavy Metal Contaminated Soil (HMCS)	
AN1. I feel calm. (reverse)	0.613
AN2. I feel secure. (reverse)	0.543
AN3. I am tense.	0.573
AN4. I am regretful.	0.529
AN5.I feel at ease. (reverse)	0.561
AN6. I feel upset.	0.666
AN7. I am presently worrying over possible misfortunes.	0.555
AN8. I feel rested. (reverse)	0.497
AN9. I feel anxious.	0.587
AN10. I feel comfortable. (reverse)	0.714
AN11. I feel self-confident. (reverse)	0.665
AN12. I feel nervous.	0.433
AN13. I am jittery.	0.643
AN14. I feel “high strung”.	0.698
AN15. I am relaxed. (reverse)	0.620
AN16. I feel content. (reverse)	0.533
AN17. I am worried.	0.657
AN18. I feel over-excited and “rattled”.	0.700
AN19. I feel joyful. (reverse)	0.419
AN20. I feel pleasant. (reverse)	0.624
Optimism (OP) (alpha = 0.738)	
OP1. In uncertain times, I always expect the best.	0.600
OP2. It is easy for me to relax.	0.879
OP3. If something can go wrong for me, it will. (reverse)	0.482
OP4. I am always optimistic about my future.	0.628
OP5. I enjoy my friends a lot.	0.711
OP6. It is important for me to keep busy.	0.676
Pessimism (PE) (alpha = 0.757)	
PE1. I hardly ever expect things to go my way.	0.485
PE2. I don’t get upset too easily. (reverse)	0.726
PE3. I rarely count on good things happening to me.	0.481
PE4. Overall, I expect more good things to happen to me than bad.(reverse)	0.733
Perceived risk (PR)(alpha = 0.851)	
To what extent do you feel that…	
PR1. …heavy metal soil contamination is caused by humans?	0.533
PR2. …HMCS may cause immediate harm to humans? (reverse)	0.502
PR3. …HMCS may cause immediate harm to you? (reverse)	0.505
PR4. …HMCS is strange to you?	0.573
PR5. …HMCS is familiar to you? (reverse)	0.576
PR6. …the government can control heavy metal soil contamination? (reverse)	0.485
PR7. …you can avoid harm from HMCS? (reverse)	0.519
PR8. …HMCS makes you worried?	0.564
PR9. …HMCS may result in harm to human health and the environment?	0.531
PR10. …HMCS may result in harm to your health?	0.680
PR11. …harm to you from HMCS is great?	0.565
PR12. …harm to humans from HMCS is great?	0.606
Social trust (ST) (alpha = 0.814)	
Considering how to solve HMCS problems, I trust information provided by…	
ST1. …the government.	0.610
ST2. …social organizations.	0.765
ST3. … experts.	0.707
ST4. …friends and relatives.	0.676
Actual knowledge (KN) (alpha = 0.777)	
From an objective perspective, HMCS results in…	
KN1. …water pollution.	0.486
KN2. …reduced soil quality.	0.531
KN3. …endangerment to human health.	0.696
KN4. …harm to livestock.	0.722
KN5. …harm to crops.	0.507
